# The Effects of *SNCA* rs894278 on Resting-State Brain Activity in Parkinson’s Disease

**DOI:** 10.3389/fnins.2019.00047

**Published:** 2019-02-04

**Authors:** Kailin Zhang, Yan Tang, Li Meng, Liping Zhu, Xiaoting Zhou, Yuwen Zhao, Xinxiang Yan, Beisha Tang, Jifeng Guo

**Affiliations:** ^1^Department of Neurology, Xiangya Hospital, Central South University, Changsha, China; ^2^School of Information Science and Engineering, Central South University, Changsha, China; ^3^Department of Radiology, Xiangya Hospital, Central South University, Changsha, China; ^4^National Clinical Research Center for Geriatric Disorders, Xiangya Hospital, Changsha, China; ^5^Key Laboratory of Hunan Province in Neurodegenerative Disorders, Central South University, Changsha, China; ^6^Center for Medical Genetics, School of Life Sciences, Central South University, Changsha, China; ^7^Parkinson’s Disease Center of Beijing Institute for Brain Disorders, Beijing China; ^8^Collaborative Innovation Center for Brain Science, Shanghai, China

**Keywords:** Parkinson’s disease, *SNCA*, resting state functional MRI, ALFF, brain activity

## Abstract

The pathogenesis of Parkinson’s disease (PD) is not well established. The rs894278 polymorphism of *SNCA* has been associated with PD. We performed this study to investigate the relationship between rs894278 and PD status on resting-state brain activity, by analyzing the amplitude of low-frequency fluctuation (ALFF). A total of 81 PD patients and 64 healthy controls were recruited. Disease severity and PD stage were evaluated in PD patients using the unified Parkinson’s disease rating scale (UPDRS) and the Hoehn and Yahr (HY) scale, while the cognitive function of all participants was assessed using the mini-mental state examination (MMSE). All participants were genotyped for the rs894278 SNP and underwent a resting state functional magnetic resonance imaging scan. We found that the ALFF values of PD patients in the lingual gyrus and left caudate were lower than those of HCs; and the ALFF values for the right fusiform of participants with G allele were lower than those of participants without G allele. And we further revealed higher ALFF values in bilateral fusiform in rs894278-G carriers than in rs894278-G non-carriers in the PD group and lower ALFF values in bilateral fusiform in rs894278-G carriers than in rs894278-G non-carriers in the HC group. Our findings show that rs894278 and PD status interactively affect the brain activity of PD patients and HCs, and changes in the brain connectomes may play a key role in the pathogenesis of PD. Thus, our work sheds light on the mechanism underlying PD pathogenesis.

## Introduction

Parkinson’s disease is a common neurodegenerative disease that affects 1.7% of Chinese population over the age of 65 (Zhang et al., 2005). The clinical manifestation of PD involves impairment of normal motor functions, such as bradykinesia, rigidity, resting tremor, and postural instability (Goedert et al., 2013). Additionally, PD is associated with non-motor symptoms that indicate multi-system impairment, such as autonomic dysfunction, sensory dysfunction, sleep disorders, and cognitive impairment (Yang et al., 2016). The hallmark pathology of PD is characterized as the loss of dopaminergic neurons in the substantia nigra pars compacta and neurite terminals projecting to the striatum, as well as the emergence of LBs in the remaining neurons (Dickson, 2012). By the time symptoms manifest enough for patients to seek PD diagnoses, striatal dopamine levels have already been reduced by over 50% (Rajput et al., 2008).

The etiology of PD remains unclear; however, it is understood that a complex interaction between genetics, environmental factors, and aging are responsible for disease development and progression. Genetic factors remain the focus of current research and, among all of the genetic factors related to PD, only a very small percentage follow monogenic inheritance patterns (Karimi-Moghadam et al., 2018). The remaining polymorphisms either do not directly contribute to PD and work in concert with other genetic variants or explain only a minute portion of disease pathology. In both cases, identified variants maintain the ability to be protective against or contribute to PD pathology (Guo et al., 2015). *SNCA*, the first gene discovered to have a causative link to PD, is located on 4q22.1 and encodes the protein a-synuclein. A-synuclein is abundant in the brain and is thought to regulate dopamine release and transport, induce fibrosis of the microtubule-associated protein tau, and reduce the reactivity of neurons to various apoptosis stimuli. Furthermore, LBs, important pathological markers of PD, are formed by the abnormal accumulation of a-synuclein (Deng and Yuan, 2014). Mounting evidence implicates genetic variants in *SNCA* to play an important role in the pathogenesis of PD. Rare mutations in *SNCA* can cause PD that follows Mendelian inheritance patterns, but this does not appear to be a common cause of PD in the Han population (Deng et al., 2015). However, many common variants of *SNCA*, including rs894278, contribute to PD risk and help to explain the appearance of PD in non-mendelian patterns (Xu W. et al., 2015). The rs894278 SNP was first found to be associated with PD through a genome-wide association study (Satake et al., 2009), and subsequent case-control studies in several independent Han populations have confirmed its impact on PD (Tan et al., 2010; Chang et al., 2011; Liu et al., 2013). Although a strong link between rs894278 and PD has been uncovered, the mechanism governing the impact of rs894278 on PD has yet to be elucidated.

Resting-state functional magnetic resonance imaging is a non-invasive quantitative brain MRI technique that explores the brain’s functional activity by analyzing changes in BOLD signals in resting individuals (Fox and Raichle, 2007). This technique has been widely applied in PD research. ALFF analysis was first proposed by Zang et al. (2007), and primarily focuses on the magnitude of low-frequency oscillations associated with hemodynamic activity. Studies of rs-fMRI in PD patients have demonstrated that changes in ALFF values in certain brain regions can effectively distinguish PD patients from normal controls, suggesting that ALFF value may be utilized as a diagnostic biomarker for PD (Skidmore et al., 2013b; Tang et al., 2017a). Furthermore, additional studies have shown that ALFF alteration is also correlated with PD symptoms such as apathy, depression, and PD motor symptoms (Skidmore et al., 2013a; Chen et al., 2015).

Depending on their location and context, genetic variants affects cellular function and in turn can manifest as alteration in normal brain structure and function. When variants present in a manner that influences brain activity, these changes can be reliably measured by fMRI. With this knowledge, a new strategy has been developed that combines genetic polymorphism information with rs-fMRI images to investigate alterations in brain activity and how variants contribute to disease state. This technique is a very sensitive and efficient way to characterize the influence of a particular SNP on brain activity and has previously been applied to neurological and psychological diseases such as schizophrenia (Wei et al., 2013), major depressive disorder (Wang et al., 2017), and Alzheimer’s disease (Wang et al., 2014). The sensitivity of this technique enables not only the detection of brain areas affected by associated SNP, but also provides enough resolution to decipher alterations in brain network architecture (Tang et al., 2017b). Information on which networks and how they are altered provides insights into how specific SNPs lead to disease phenotypes and the mechanisms that govern phenotypic changes. A recent study conducted by Nombela et al. (2014) applied this strategy to PD using task fMRI and found that rs4680 of *COMT* gene, rs9468 of *MAPT* gene, and rs429358 of *APOE* gene modulated different brain areas each associated with different cognitive domains. Additionally, brain function is altered before clinical symptoms present. Thus, the ability to genotype individuals and detect altered brain activity (or fMRI images) characteristic of PD can be used as a powerful tool for early diagnosis before other clinically diagnosable features emerge (van Nuenen et al., 2009).

In this study, we speculate that the genotype of the rs894278 SNP influences the brain activity of PD patients and is involved in the pathogenesis of PD. We utilize the strategy outlined above to explore the differential brain activity of PD patients and HCs with different *SNCA* rs894278 genotypes.

## Materials and Methods

### Participants

A total of 81 PD patients and 64 age and sex matched HCs were recruited at Xiangya Hospital, Central South University, from April 1, 2013 to February 26, 2017. PD diagnosis was made, according to the UK PD Society Brain Bank clinical diagnostic criteria (Hughes et al., 1992), by two experienced neurologists. All participants were right-handed Chinese individuals without any other organic diseases, mental disorders or contraindications to MRI scanning. In addition, all patients were free from antiparkinsonian drugs for at least 12 h before the clinical and MRI assessments.

This study was approved by the Xiangya Hospital ethics committee, and written informed consent was provided by every participant prior to beginning this study.

### Clinical Assessment

Disease severity and PD stage were evaluated in PD patients using the UPDRS and the HY scale, while the cognitive function of all participants was assessed using the MMSE. The tremor, rigidity, bradykinesia, and posture/gait score were calculated by the sum of scores from item 20 + 21, item 22, item 23 + 24 + 25 + 26 + 31, and item 27 + 28 + 29 + 30 of UPDRS, separately. In addition, the LEDD was computed based on existing methods (Tomlinson et al., 2010), namely: total LEDD = immediate release L-dopa dose × 1 + L-dopa × 0.33 (concurrent entacapone ingestion) + controlled release L-dopa dose × 0.75 + duodopa × 1.11 + pramipexole × 100 + selegiline × 10 + rasagiline × 100 + piribedil × 1 + amantadine × 1 + ropinirole × 20 + rotigotine × 30. MMSE scores between PD patients and HCs were compared, while UPDRS, HY, and LEDD scores were compared between different PD patient groups.

### Genotyping

Genomic DNA of all participants was extracted from peripheral blood samples using standard phenol–chloroform procedures. The SNP rs894278 was genotyped using time-of-flight mass spectrometry. Every participant was either classified as a G carrier or G non-carrier after genotyping.

### MRI Data Acquisition

MRI data were acquired using a 3.0T GE Signa MR scanner (General Electric, Fairfield, CT, United States) in the Department of Radiology of Xiangya Hospital, Central South University. Resting-state functional images covering the whole brain were recorded using a gradient EPI sequence with the following parameters: TR = 2,000 ms, TE = 30 ms, voxel size = 3.44 × 3.44 × 4.60, flip angle = 90°, slice number/thickness = 32/4.0 mm. Every participant was asked to rest calmly and keep his or her head still during the MRI scan. During a 6-min time period, 180 time points were obtained for each participant.

### MRI Data Preprocessing

MRI data were preprocessed with the DPARSF^1^, which is based on SPM version 8^2^. The procedures used were as follows: From the total of 180 slices of rs-fMRI data from each participant, the first 10 were removed, and the remaining 170 slices were corrected. Head motion of each slice was realigned, while data from participants with head motion exceeding 1.5 mm or 1.5° were excluded. The slices were normalized to the MNI template after dimension changes and the quality of resultant images were manually checked. Next, smoothing was carried out using a 4 × 4 × 4 FWHM Gaussian kernel. Following smoothing, linear and quadratic trends resulting from the MRI machine, motion, or noise were removed. Finally, signals of head parameter in six dimensions, global brain, white matter and cerebrospinal fluid were regressed out. After data preprocessing, data from 15 PD patients and 6 HCs were excluded from the study, thus 66 PD patients and 58 HCs remained for the final analyses.

### ALFF Analysis

To remove very-low-frequency drift and high frequency noise, time series between 0.01 and 0.08 Hz of each voxel were extracted and converted to frequencies using a Fast Fourier Transform and the power spectrum was subsequently obtained. The power spectrum’s square root was calculated and is referred to as the ALFF value. The ALFF value of each voxel was then divided by the mean ALFF of the whole brain to produce the mALFF. ALFF values between different groups were compared and significant clusters were obtained and shown using Automated Anatomical Labeling (AAL) brain atlas. Finally, ALFF values of clusters showing significant differences were correlated with clinical assessment scores, such as the UPDRS, UPDRS I, UPDRS II, UPDRS III, UPDRS IV, tremor, rigidity, bradykinesia, and posture/gait scores, HY scale stage, and LEDD, for PD patients who were rs894278-G carriers.

### Statistical Analysis

Demographic and clinical data between PD patients and HCs, as well as clinical data between different groups of PD patients, were analyzed using SPSS version 21.0 (SPSS Inc., Chicago, IL, United States). For continuous variables, analyses were performed using a Student’s *t*-test or Mann–Whitney *U* test. For categorical variables, analyses were performed using χ^2^ tests. The statistical significance threshold was defined as *P* < 0.05.

MRI data between groups were analyzed and compared with SPM8. Full factorial analysis was conducted to analyze the influences of diagnosis and genotype on rs-fMRI images, through which the independent and interactive effects of diagnosis and genotype were obtained in all participants. For areas with a significant diagnosis × genotype interaction effect, *post hoc* analyses were subsequently conducted to separately investigate the genotype effect in the PD and HC groups. The statistical significance threshold was defined as *P* < 0.01 (AlphaSim corrected). Correlation analysis between ALFF values and clinical data was performed using partial correlation analysis to remove potential confounding MMSE scores (FDR corrected).

## Results

### Demographic and Clinical Data

There were no significant differences in age, sex or genotype between the PD and HC groups. However, the MMSE scores were lower in the PD group than in the HC group (Table 1).

**Table 1:** Demographic and clinical data between PD and HC groups.

Characteristics	PD (*n* = 66)	HC (*n* = 58)	*P*
Age (years)	53.76 ± 10.72	51.67 ± 10.80	0.284
Sex (male/female)	35/31	29/29	0.736
Genotype (G carriers/G non-carriers)	41/25	33/25	0.554
MMSE	26.78 3.65	27.88 ± 3.31	0.023^∗^
UPDRS score	49.02 ± 22.05	–	–
UPDRS I score	3.00 ± 1,92	–	–
UPDRS II score	13.29 ± 6.48	–	–
UPDRS III score	30.67 ± 15.26	–	–
UPDRS IV score	2.06 ± 3.22	–	–
Tremor score	4.62 ± 3.62	–	–
Rigidity score	5.71 ± 4.26	–	–
Bradykinesia score	13.09 ± 7.17	–	–
Posture/gait score	4.83 ± 2.89	–	–
HY scale	2.33 ± 0.77	–	–


*Data are shown as the mean ± standard deviation. “G carriers” represents the participants who possess at least one rs894278 G allele; “G non-carriers” represents the participants who possess no rs894278 G alleles. PD, Parkinson’s disease; HC, healthy control; MMSE, mini-mental state examination. ^∗^*P <* 0.05.*

There were no significant differences in the age of onset, course of disease, scores for UPDRS, UPDRS I, UPDRS II, UPDRS III, UPDRS IV, tremor, rigidity, bradykinesia, or posture/gait, HY scale stage, or LEDD between rs894278-G carriers and non-carriers among PD patients (Table 2).

**Table 2:** Clinical data between rs894278-G carriers and non-carriers among PD patients.

Characteristics	G carriers (*n* = 41)	G non-carriers (*n* = 25)	*P*
Age of onset	48.37 ± 12.21	46.72 ± 11.89	0.547
Course of disease	5.41 ± 4.26	7.00 ± 6.53	0.801
UPDRS score	47.90 ± 19.77	50.84 ± 25.70	0.603
UPDRS I score	3.20 ± 1.72	2.68 ± 2.21	0.133
UPDRS II score	13.07 ± 5.70	13.64 ± 7.71	0.968
UPDRS III score	29.85 ± 2.27	32.00 ± 16.59	0.583
UPDRS IV score	1.78 ± 3.21	2.52 ± 3.25	0.239
Tremor score	4.22 ± 3.64	5.28 ± 3.54	0.193
Rigidity score	5.54 ± 4.62	6.00 ± 3.67	0.672
Bradykinesia score	12.95 ± 6.57	13.32 ± 8.18	0.841
Posture/gait score	4.71 ± 2.87	5.04 ± 2.96	0.557
HY scale	2.34 ± 0.69	2.32 ± 0.90	0.967
LEDD	421.25 ± 247.09	391.00 ± 312.84	0.330


*Data are shown as the mean ± standard deviation. “G carriers” represents the participants who possess at least one rs894278 G allele; “G non-carriers” represents the participants who possess no rs894278 G alleles. PD, Parkinson’s disease; UPDRS, unified Parkinson’s disease rating scale; HY, Hoehn and Yahr; LEDD, levodopa equivalent daily dose.*

### Group Based Differences in ALFF

**FIGURE 1:**
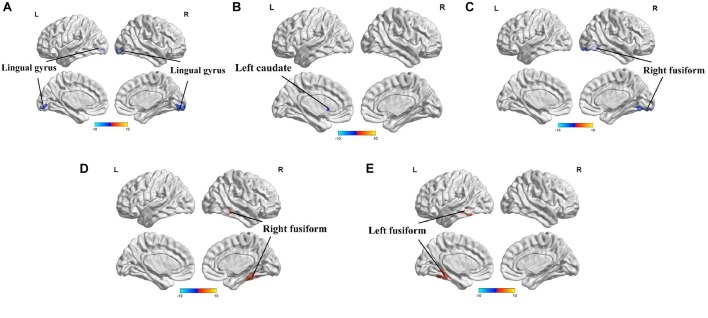
Amplitude of low-frequency fluctuations (ALFF) analysis of resting-state brain activity for G carriers vs. non-carriers of the rs894278 genotype in the Parkinson’s disease (PD) and healthy control (HC) groups. **(A)** ALFF at the lingual gyrus in PD patients is decreased relative to HCs. **(B)** ALFF at the left caudate in PD patients is decreased relative to HCs. **(C)** ALFF at the right fusiform in rs894278-G carriers is decreased relative to G non-carriers. **(D)** ALFF comparisons for the right fusiform in PD patients and HCs for rs894278-G carriers and G non-carriers. **(E)** ALFF comparisons for the left fusiform in PD patients and HCs for rs894278-G carriers and G non-carriers.

**FIGURE 2:**
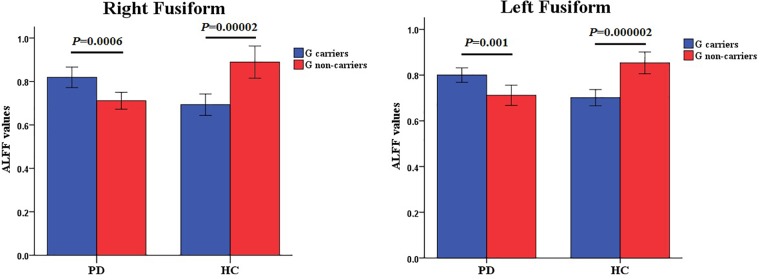
Diagnosis × genotype effects on ALFF values. The color bar shows *post hoc* comparisons in the clusters with significant diagnosis × genotype effects. The differences between rs894278-G carriers and non-carriers in the PD or HC group were significant separately. Data are shown as the mean ± standard error. ALFF, amplitude of low-frequency fluctuations; PD, Parkinson’s disease; HC, healthy control.

The full factorial analysis model revealed significant diagnosis effects in the lingual gyrus and left caudate, genotype effects in the right fusiform, and diagnosis × genotype effects in the right and left fusiform (Table 3 and Figure 1). As depicted in Figure 1, the ALFF values of PD patients in the lingual gyrus and left caudate were lower than those of HCs; and the ALFF values for the right fusiform of participants with G allele were lower than those of participants without G allele. A *post hoc* analysis of clusters with significant diagnosis × genotype effects further revealed higher ALFF values in G carriers than in non-carriers in the PD group and lower ALFF values in G carriers than in non-carriers in the HC group (Figure 2).

**Table 3:** ALFF comparison of rs894278 between the PD and HC groups with different rs894278 genotypes.

Regions (AAL)	Number of voxels	Peak MNI coordinates			*T*-value	*P*
		*X*	*Y*	*Z*		
Diagnosis effects						
Lingual gyrus	138	3	-96	-12	-5.7379	<0.01
Caudate_L	129	-12	12	3	-4.9698	<0.01
Genotype effects						
Fusiform_R	176	30	-63	-3	-4.5923	<0.01
Diagnosis × genotype effects						
Fusiform_R	121	30	-33	-24	4.485	<0.01
Fusiform_L	106	-30	-39	-21	4.1886	<0.01


*The *T*-value denotes the statistical value of the peak voxel. ALFF, amplitude of low-frequency fluctuations; PD, Parkinson’s disease; HC, healthy control; MNI, Montreal Neurological Institute; L, left; R, right*.

### Correlation Analysis

After removing confounding MMSE scores, we found a significant negative correlation between UPDRS score and ALFF values in the lingual gyrus that showed a diagnosis effect (*P* = 0.049, *r* = -0.322) and a significant negative correlation between UPDRS I score and ALFF values in the lingual gyrus that showed a diagnosis effect (*P* = 0.028, *r* = -0.358) in PD patients carrying the rs894278 G allele. We also discovered a significant positive correlation between posture/gait scores and ALFF values in the left fusiform that showed a diagnosis × genotype interaction effect (*P* = 0.008, *r* = 0.548) and a significant positive correlation between HY scale stage and ALFF values in the left fusiform that showed a diagnosis × genotype interaction effect (*P* = 0.008, *r* = 0.548) in PD patients without rs894278 G allele. Unfortunately, we did not find any significant correlation between ALFF values in the brain regions that showed group-based differences and clinical assessments after multiple test correlation.

## Discussion

This is the first study to investigate the effect of *SNCA* rs894278 on ALFF in PD patients and HCs. Our results show that the ALFF values of PD patients in the lingual gyrus and the left caudate were lower than those of HCs, and that the ALFF value of participants with G allele in the right fusiform were lower than those of participants without G allele. We further reveal higher ALFF values in rs894278-G carriers than in rs894278-G non-carriers in the PD group and lower ALFF values in rs894278-G carriers than in rs894278-G non-carriers in the HC group. These findings provide strong evidence to support the notion that *SNCA* rs894278 works in conjunction with PD status to affect brain activity in an interactive manner in PD patients and HCs.

The significant diagnosis × genotype effect in the bilateral fusiform means that the function of the bilateral fusiform gyrus was influenced by both the diagnosis of PD and the rs894278 genotype. Furthermore, PD patients with the rs894278 G allele had higher spontaneous brain activity than those without the G allele in the bilateral fusiform gyrus, while HCs with the rs894278 G allele had lower spontaneous brain activity than HCs without the G allele in these regions. This indicates that the presence of a rs894278 G allele exacerbates PD symptoms though alterations in the fusiform structure and function. The fusiform plays many roles in the proper function in several neurological pathways and contributes to cognitive function, facial recognition, the visual pathway, and autonomic function. With respect to the ventral visual pathway, irregularities in the fusiform is tightly linked to dysfunction of visual processing and can lead to hallucinations in PD patients (Oishi et al., 2005). Brain GMV in the fusiform of PD patients is smaller than that of HCs, and the decreased GMV in this region is associated with worse cardiovascular autonomic function (Chen et al., 2016). In the fusiform gyrus of PD patients with and without MCI, longitudinal BOLD signals are reduced in patients with MCI relative to those free from cognitive impairment (Ekman et al., 2014). In addition, Rypma et al. (2015) tested the dopamine D1 BP using PET and BOLD signal change via fMRI when the participants were performing a face recognition task; the results showed that the D1 BP and BOLD signal in the fusiform gyrus were significantly related. Taken together, this indicates that the fusiform gyrus affects non-motor functions of PD, and the activity of the fusiform can be regulated by dopamine. We found that PD diagnosis and rs894278 genotype can influence fusiform activity, hinting at a link between rs894278 genotype and the non-motor functions of PD. This finding is consistent with a recent study showing that the rs894278 genotype is associated with hyposmia in PD (Li et al., 2017). However, it is difficult to tell whether the dysfunction in the fusiform is caused by PD itself, a compensatory mechanism of the disease, or chronic dopaminergic treatment. Further investigation would be necessary to determine exact underpinnings responsible for fusiform dysfunction in PD patients.

Furthermore, when we divided all participants into two groups according to rs894278 genotype, participants with the rs894278 G allele had lower brain activity compared to those without the rs894278 G allele in the right fusiform. However, PD patients with the rs894278 G allele had higher brain activity than those without the rs894278 G allele. Thus, the effect of rs894278 genotype on brain activity is reversed in the PD group. These data indicate that the fusiform may be a key brain region where the rs894278 polymorphism has an impact on PD pathogenesis. As depicted above, the fusiform is associated with cognitive function and therefore the rs894278 SNP is likely associated with the cognitive impairment seem in PD patients. Prior research has shown that genetic variation of the *SNCA* gene is associated with dementia in PD (Guella et al., 2016). This previous link between a gene variant and dementia in PD has been characterized, gives weight to our hypothesis that rs894278 SNP is linked to the decline of cognitive function in PD. This evidence indicates that rs894278 is an interesting candidate for future validation with functional studies. Our results also show that PD patients and HCs exhibit different brain activity in the left caudate and lingual gyrus, which are associated with the motor (Tessitore et al., 2012; Xu Y. et al., 2015) and non-motor functions of PD (Niethammer et al., 2013; Jung et al., 2014; Kiferle et al., 2014; Liu et al., 2015), respectively. Thus, we speculate that there is a relationship between the fusiform, caudate, and lingual areas, and the interaction of these regions influences the integrated brain activity of patients and controls. Tan et al. (2011) stated that the fusiform, caudate, and lingual engaged in language learning, and Ulrich and Kiefer (2016) showed that the caudate and ventral visual stream areas (which include the fusiform and lingual gyri) have altered connections in subliminal visuomotor priming. These studies found that the fusiform, caudate and lingual work together in a certain brain activity, which support the idea of the potential relationship between these brain regions.

Although our findings are robust, there are some limitations to our study. First, cognitive status was not well matched between our PD and HC groups, so results may be affected by interference from regions related to cognitive function. In addition, although the average MMSE scores of the two groups were both in the normal range, the score was slightly lower in the PD group. This suggests that PD patients may already have had some cognitive impairment even though their MMSE scores showed their cognitive function to be ‘normal.’

## Conclusion

In conclusion, we investigated the effect of *SNCA* rs894278 on ALFF in PD patients and HCs and found altered spontaneous activity in certain brain regions that were influenced by PD status and rs894278 genotype. The results indicate that rs894278 affects the occurrence of PD. Furthermore, changes in the brain connectomes may play a key role in the pathogenesis of PD. Overall, our work sheds light on the mechanisms underlying PD pathogenesis.

## Author Contributions

KZ, YT, BT, and JG conceived the study. KZ and YT analyzed the fMRI data. LM, LZ, XZ, and YZ collected the fMRI data. LZ, XZ, YZ, and XY collected the clinical data. KZ drafted the manuscript. YT revised the manuscript. BT and JG supervised the study and obtained funding. All authors have approved the final article.

## Conflict of Interest Statement

The authors declare that the research was conducted in the absence of any commercial or financial relationships that could be construed as a potential conflict of interest.

## Footnotes

1 http://www.restfmri.net

2 https://www.fil.ion.ucl.ac.uk/spm/

## Abbreviations

ALFF: amplitude of low-frequency fluctuation
BOLD: blood oxygen level dependent
BP: binding potential
DPARSF: Data Processing Assistant for Resting-State fMRI software
EPI: echo planar imaging
FWHM: full width at half maximum
GMV: gray matter volume
HCs: healthy controls
HY: Hoehn and Yahr scale
LBs: Lewy bodies
LEDD: levodopa equivalent daily dose
MCI: mild cognitive impairment
MMSE: mini-mental state examination
MNI: Montreal Neurological Institute
PD: Parkinson’s disease
PET: positron emission tomography
rs-fMRI: resting-state functional magnetic resonance imaging
SNP: single nucleotide polymorphism
SPM: Statistical Parametric Mapping
TE: echo time
TR: repetition time
UPDRS: unified Parkinson’s disease rating scale
